# Risk Practices for Occupational Zoonotic Exposure to Tuberculosis in a High-Risk Population in Portugal

**DOI:** 10.3390/tropicalmed8030167

**Published:** 2023-03-13

**Authors:** Ana Carolina Abrantes, João Serejo, Madalena Vieira-Pinto

**Affiliations:** 1 CECAV-Animal and Veterinary Research Centre, UTAD, Quinta de Prados, 5000 Vila Real, Portugal; 2 Câmara Municipal de Idanha-a-Nova, 6060 Idanha-a-Nova, Portugal; 3 Department of Veterinary Sciences, Trás-os-Montes e Alto Douro University, Quinta de Prados, 5000 Vila Real, Portugal; 4 AL4AnimalS-Associate Laboratory for Animal and Veterinary Sciences, 5000 Vila Real, Portugal

**Keywords:** hunters, hunting, initial examination, large game, public health, tuberculosis-like lesions

## Abstract

Concerning large game in Portugal, there is an epidemiological risk area for tuberculosis where the pressure of tuberculosis infection in wild animals is high. Hunters and other people involved in managing carcasses (evisceration and/or initial examination) of these animals should be considered as a high-risk population for sporadic occupational zoonotic infection. This study aims to evaluate and indicate these stakeholders’ main risk practices. The survey was carried out in two phases: a first phase with an anonymous survey to hunters about self-consumption of game meat and carcass handling practices, and a second phase of evaluation *in loco* of the practices carried out at collection points after driven hunts. As the main results suggested in this study, bad practices and improper handling of hunted carcasses potentially infected with tuberculosis were marked in both phases of the survey, mostly related to the lack of recognition of tuberculosis-like lesions and the non-use of individual protection material, such as gloves and masks. It is evident that stakeholders are interested in learning more about how to perform initial examination properly and what kind of biosecurity measures can be adopted to minimize zoonotic infection risk.

## 1. Introduction

Tuberculosis (TB) is one of the most infectious diseases, with a great impact worldwide. In developed countries in the past decades, for eradicating TB, the “test-and-slaughter” strategy in livestock and the pasteurization of milk have dramatically reduced the risk of zoonotic infection [1]. However, this risk cannot be underestimated/overlooked in specific high-risk areas, specifically those with marked populations in daily contact with animals, their derived products, and their carcasses. In these cases, potential zoonotic tuberculosis infection can be considered a major public health issue [1,2,3].

Like other developed countries, for decades, Portugal has had a TB eradication program for livestock, making it possible to greatly reduce the impact of zoonotic TB infection that results from contact with these animals. With the proliferation of extensive production, animal TB is nowadays a problem not restricted to domestic animals but also wild fauna as well [3]. After years of revising TB in the wild population, it is known that species such as wild boar (*Sus scrofa*) and red deer (*Cervus elaphus*) act as hosts and disseminators of the disease. The wild boar is even considered to be its sentinel [4]. Coincidentally, these are also the two main large game species hunted in Portugal, thus causing hunters and other players in the hunting processes to be in greater exposure to these species than any other wild species [5].

In wild fauna, no TB eradication strategy has ever been implemented [6]. In Portuguese territory, the evidenced presence of TB agent in large game animals constitutes a risk factor that encumbers the eradication and control of bovine TB and increases public health concerns [3,4,7]. Laboratory testing data show that about half of the samples of lesions compatible with tuberculosis collected in wild boar (48.6%) and deer (47.7%) after sanitary evaluation were caused by *Mycobacterium bovis* and *Mycobacterium caprae* [7]. In Portugal, in 2011, a Notice Nº1 of tuberculosis in large game was implemented that delimits an “epidemiological risk area for tuberculosis for the large game” [3,7,8]. The main objectives of implementing this Notice Nº1 as well as specific rules in the delimited area were: (i) health protection of game handlers and hunters themselves; (ii) systematic and mandatory initial examination of game pieces, performed by a veterinarian, that are intended for self-consumption or placing on the market; (iii) correct routing and disposal of by-products [9]. Some specific hygiene conditions are also required for collection points (place of evisceration/initial examination) in hunting areas located in a specific risk area, such as: (i) devices for protection from the weather and for waterproofing the soil, (ii) adequate lighting, drinking water, disinfection material and proper knives. Personal protective equipment, such as disposable gloves and masks, should also be made available on-site [9,10,11].

In an area where TB agents circulate in larger game species, carcasses handled in post-driven hunts can pose a risk of exposure to hunters’ and game handlers’ health. All rules and requirements for hygienic and sanitary conditions at the place of evisceration and initial examination of large game carcasses are crucial to minimizing the risk of contamination and zoonotic exposure [10,11]. The initial examination must allow the evaluation of different organs and thus, in risk areas defined in Notice Nº1, identifying tuberculosis-like lesions (TBL) at marked points of the infected carcasses [3,7,12]. These lesions, compatible with some zoonotic infectious agents, could be a source of infection by direct contact, aerosols and cutting knives that release the infectious agents encapsulated in lesions [11,13].

To understand the reality in the field of implementing rules and good practices indicated in Notice Nº1 and the correct identification of TBL with zoonotic potential, as well as the perception of hunters regarding these issues for their health, this study was carried out. The main objective was to assess which bad practices for occupational zoonotic exposure of TB were the main risks for these high-risk stakeholders (hunters and game handlers mainly), carried out in the epidemiological risk area for tuberculosis for large game in Portugal.

## 2. Materials and Methods

To assess the risk of bad practices carried out by hunters when eviscerating and performing the initial examination of large game carcasses potentially infected with TB, a survey was carried out in two phases: i. a first phase with an anonymous survey to hunters about self-consumption of game meat and good carcass handling and hygiene practices and ii. a second phase of evaluation *in loco* of practices carried out at collection points after driven hunts in the epidemiological risk area for tuberculosis from large game, where it is mandatory to eviscerate and perform the initial examination on-spot before translocating the carcasses out of the collection points.

### 2.1. First Stage: Random Survey of Hunters on Self-Consumption and the Perceived Risk Practices for Tuberculosis

In this first phase, a survey entitled “Self-consumption of game meat and hygiene practices” was distributed to hunters who practice their hunting activity at least once a year in the area marked as an epidemiological risk by Notice Nº1 who had contact with the carcasses or even self-consumed game meat slaughtered in that area. 

The data are not clear, but it is known that around 15,000 hunters in Portugal are licensed to hunt in the hunting region that encompasses the epidemiological risk area for tuberculosis in large game. The sample (*n* = 106) was random and the objective was to reach as many hunters as possible during the 2020/21 and 2021/22 hunting seasons. The survey was distributed randomly during the driven hunts and all hunters filled it out willingly. Personal data were not collected and informed consent was obtained from all hunters.

The relevant questions for assessing the risk of bad practices during the handling and hygiene of large game carcasses potentially infected with TB, which lead to a greater risk of zoonotic occupation exposure, were the following:

- Have you taken the course “Lage game initial examination for hunters”? Yes/No
- Do you carry out the evisceration and/or initial examination of large game carcasses for self-consumption? Yes/No/Sometimes
- During evisceration and/or initial examination, do you use individual protection measures, such as the use of gloves? Always/Never/Sometimes
- Have you ever seen this type of lesion during the evisceration of a carcass (purulent material in the lymph nodes of the head near the neck and/or in the lymph nodes of the intestine)? (question accompanied by an image of a Tuberculosis-like lesion) Yes/No
- When you usually see this type of lesion, how do you usually proceed? Discard only the affected parts/Discard the complete carcass/I don’t think any extra action is necessary and continue the evisceration as normal/Non-applicable
- When knives touch these lesions as seen in the previous image, do you have the habit of always disinfecting knives before starting the evisceration of the next carcass? Always/Never/Sometimes/Non-applicable

### 2.2. Second Stage: Evaluation in Loco of the Practices Carried out at Collection Points

**Figure 1:**
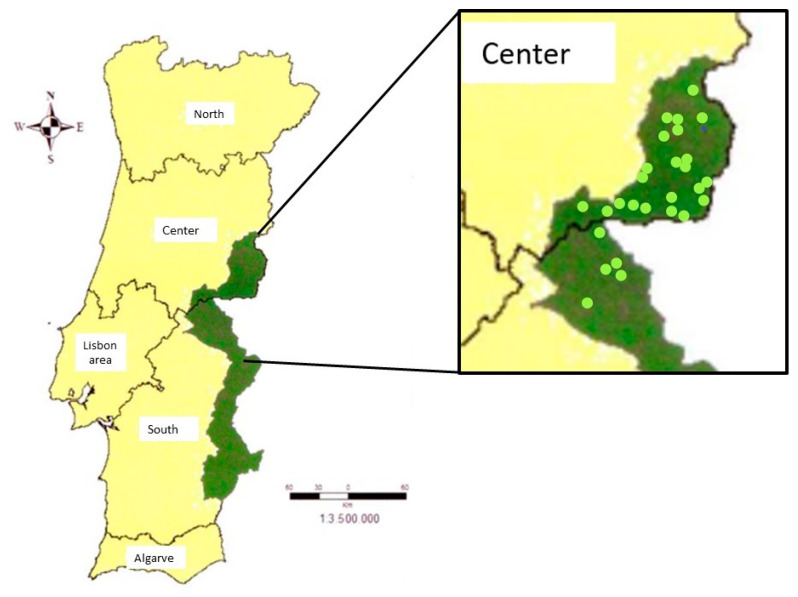
Marked (dark green) epidemiological risk area for tuberculosis for large game in Portugal (Notice Nº1) and in image enlargement, the 27 collection points assessed in this study (light green points).

During two hunting seasons (2021/22 and 2022/23), 27 randomly evaluated collection points were all located within the perimeter delimited as an epidemiological risk area for tuberculosis for large game in Portugal (Figure 1), which was chosen due to the high probability of finding carcasses with TB infection. At this stage of the work, practices that may pose a risk of exposure to TB on the part of hunters were evaluated/marked. Thus, the points evaluated were the following:

- Good organization (of human and material resources) during evisceration and/or initial examination: Good/Bad
- Use of personal protective equipment (specific disposable gloves…): Yes/No
- Changing protective material, specifically gloves, when in contact with Tuberculosis-like lesions? Yes/No/Non-applicable
- Use of collection points’ specific knives? Yes/No
- Systematic disinfection of knives during evisceration and/or initial examination? Yes/No/Sometimes
- Specific disinfection of knives after contact with Tuberculosis-like lesions: Yes/No/Non-applicable
- Existence of extra care with contaminated carcasses and by-products: Yes/No/Non-applicable

## 3. Results

In the period of 2021−2022, one hundred and six hunters (*n* = 106) from north to south in Portugal aged between 18 and 70 years answered the anonymous survey carried out in the first phase of this work (Table 1). Informed consent was obtained from all hunters. For the second phase, 27 collection points were visited at the end of the driven hunts, to assess *in loco* the evisceration practices and/or initial examination by the hunters and veterinarians present, always by the same researcher (Table 2). 

## 4. Discussion

It is evident that the initial examination of large game is a very useful tool in the detection of TBL. Knowing how to distinguish lesions associated with a potential TB infection in hunted animals and knowing how to act in case they are observed is an asset to control and mitigate the spread of the disease at the wildlife–human interface. Extra care in handling carcasses and individual biosecurity measures and/or the use of equipment associated with the correct practice of evisceration as well as initial examination are primary barriers to mitigate the risk associated with this occupational exposure to TB between large game species and hunters [8,10].

The self-consumption of large game meat is a usual practice in Portugal, and more than 95% of hunters answered positively to this respective question of the survey. This practice is considered high-risk, especially when the previous initial examination of the carcasses is not carried out. This risk is associated not only with TB, but also with other foodborne zoonoses [14,15]. However, the manipulation of carcasses of large game without knowledge and individual protection equipment, especially gloves and masks, is an element that increases the risk of TB exposure and secondary infection. This transmission can occur by direct contact with lesions and/or by aerosol or contact with carcasses that have suffered cross-contamination [10;11]. In this case, in the first phase of our study, more than half of the respondents (58.5%) answered that they only use protective material, especially gloves, sometimes or even never. However, in the second phase of our study, it was found that in the on-spot observations, there was an increase in the percentage of participants wearing gloves, with gloves being used in 14 spots (51.8%). The biggest concern was that oftentimes not-disposable gloves were used. However, when a veterinarian was observed to be the one performing the initial examination on-spot, the gloves used were always disposable, but only in 26% of cases was there preoccupation about changing them after contact with carcasses with TBL to avoid cross-contamination between carcasses and other objects, as in the case of knives [8,16]. In addition, in the second phase of this work, the use of knives, their disinfection and replacement after handling/cutting TBL were evaluated to assess the risk associated with these practices. Of the 27 evaluated spots, carcasses with TBL were found in 16. However, the existence of knives specific to the spot was rare (only 1) and systematic disinfection between carcasses was scarce, occurring in only half of the collection points where TBLs were observed. The rule of disinfection after contact with a TBL is only sometimes complied with. As previously stated, this is a major risk factor since there is cross-contamination of carcasses and dispersion and proliferation of tuberculosis bacilli both on surfaces and in the form of aerosols. This increases the risk of accidental exposure of hunters to this disease [13,17].

In short, there are many flaws in the field of preventing zoonotic exposure to TB in this specific risk population, the large game hunters [18]. Bad practices in handling hunted carcasses potentially infected with TB increase the risk of occupational exposure. It is clear from the study that stakeholders are interested in learning more about properly performing evisceration and initial examination and what biosecurity measures can be adopted to minimize this known risk [10,11]. Many hunters answered that they had never seen TBL in carcasses, not identifying the lesion on the image, thus leading to a potential misdiagnosis. Not being able to identify TBL can sometimes lead to carcasses with TBL being consumed or discarded improperly [19]. A carcass with TBL must be discarded completely and, if possible, in a by-product treatment unit [20].

Part of this study (observational evaluation *in loco*) was carried out in an epidemiological risk area of tuberculosis for large game (Notice Nº1). Since 2011, many rules must be complied with in this area to mitigate the impact of TB infection, using the initial examination as a tool for detecting TB in wildlife. Therefore, the probability of TBL being observed at the time of initial examination is greater and, thus, it is possible to analyze with greater reliability the risk practices related to the handling and cutting of TBL [3,7,9,10,11]. With the results obtained in this study, it was verified that after more than 10 years of this Notice Nº1 and its respective rules in force, many risky practices for TB zoonotic exposure and transmission are still practiced.

## 5. Conclusions

In high-risk populations for TB, such as hunters and other stakeholders who deal directly with the carcasses of potentially infected large game animals, it is necessary to assess the risk of their usual practices, especially those that are bad practices and increase the risk of exposure. The highest-risk practices should be highlighted, prioritizing those that most effectively protect hunters, with scientific data and qualitative and quantitative information [21]. In this case, the use of individual protection material, such as disposable masks and gloves, and extra care taken with contaminated knives should be highly recommended practices at all collection points. These items should be recommended not only in the epidemiological risk area but also in all Portuguese territory.

More information is further needed. The initial examination training of hunters and veterinarians must be reinforced. In addition to allowing TBL to be recognized, it also provides proactive training in compliance with biosecurity measures and reinforcement of self-protection measures to this high-risk population of hunters, veterinarians, game managers and other game handlers.

Prevention, updated information, and training are the best measures to mitigate the risk practices for occupational zoonotic exposure to TB in this high-risk population. With these measures, the objective should be that, after another 10 years of implementation of Notice Nº1, the stakeholders are aware of the bad practices of exposure to TB in this area of risk for wild animals. Thus, it should be noted that much work remains to be carried out for a full awareness of this population at risk: stakeholders related to hunting, mainly hunters.

## Figures and Tables

**Table 1:** General overview of 106 responses from a random survey of hunters on self-consumption and the perceived risk practices for tuberculosis.

Questions	Responses		
General Questions			
Have you taken the course “Lage game initial examination for hunters”?	Yes *n* = 41; 38.7%	No *n* = 50; 47.2%	No answer *n* = 15; 14.1%
Do you carry out the evisceration and/or initial examination of large game carcasses for self-consumption?	Yes *n* = 95; 89.6%	No *n* = 6; 5,7%	Sometimes *n* = 5; 4.7%
During evisceration and/or initial examination, do you use individual protection measures, such as the use of gloves?	Always *n* = 44; 41.5%	Never *n* = 37; 34.9%	Sometimes *n* = 25; 23.6%
Recognition of Tuberculosis-like Lesions			
Have you ever seen this type of lesion during the evisceration of a carcass (purulent material in the lymph nodes of the head near the neck and/or in the lymph nodes of the intestine)?	Yes *n* = 28; 26.4%	No *n* = 78; 73.6%	
When you usually see this type of lesion, how do you usually proceed?	Discard only the affected parts *n* = 7; 6.6%	Discard the complete carcass *n* = 70; 66%	Non-applicable *n* = 29; 27.4%
When knives touch these lesions as seen in the previous image, do you have the habit of always disinfecting the knives before starting the evisceration of the next carcass?	Always *n* = 72; 67.9%	Never *n* = 20; 18.7%	Non-applicable *n* = 14; 13.2%

**Table 2:** Overview of the evaluation *in loco* of the practices carried out at collection points.

Questions	Responses		
Use of Protective Material			
Good organization (of human and material resources) during evisceration and/or initial examination:	Good *n* = 22; 81.5%	Bad n = 5; 18.5%	
Use of personal protective equipment (specific disposable gloves…):	Yes *n* = 14; 51.8%	No *n* = 13; 48.2%	
Use of collection points’ specific knives?	Yes *n* = 6; 22.2%	No *n* = 21; 77.8%	
Systematic disinfection of knives during evisceration and/or initial examination?	Yes *n* = 1; 3.7%	No *n* = 18; 66.7%	Sometimes *n* = 8; 29.6%
Evaluation of collection points where occur TBL observation (*n* = 16)			
Changing protective material, specifically gloves, when in contact with Tuberculosis-like lesions?	Yes *n* = 7; 26%	No *n* = 9; 33.3%	Non-applicable *n* = 11; 40.7%
Specific disinfection of knives after contact with Tuberculosis-like lesions:	Yes *n* = 8; 29.6%	No *n* = 8; 29.7%	Non-applicable *n* = 11; 40.7%
Existence of extra care with contaminated carcasses and by-products:	Yes *n* = 8; 29.7%	No *n* = 8; 29.6%	Non-applicable *n* = 11; 40.7%
